# Complete and Assembled Genome Sequence of Salmonella enterica subsp. enterica Serotype Senftenberg N17-509, a Strain Lacking Salmonella Pathogen Island 1

**DOI:** 10.1128/genomeA.00156-18

**Published:** 2018-03-22

**Authors:** Marc J. A. Stevens, Katrin Zurfluh, Denise Althaus, Sabrina Corti, Angelika Lehner, Roger Stephan

**Affiliations:** a Institute for Food Safety and Hygiene, Vetsuisse Faculty, University of Zurich, Zurich, Switzerland

## Abstract

The genome of Salmonella enterica subsp. enterica serotype Senftenberg N17-509, a strain isolated from desiccated coconut, was sequenced using single-molecule real-time sequencing. It consists of a 5.1-Mbp chromosome and a 29-kb linear plasmid.

## GENOME ANNOUNCEMENT

The Salmonella enterica subsp. enterica serotype Senftenberg strain N17-509 was isolated from desiccated coconut and provided different identification and typing challenges. The biochemical tests performed to confirm the Salmonella genus were inconclusive. A commercial real-time PCR identification system (Assurance GDS Salmonella kit; BioControl Systems, Bellevue, WA, USA) based on the *invA* gene yielded a negative result. A further rRNA-based fast method (VIT Salmonella kit; vermicon AG, Munich, Germany) and the matrix-assisted laser desorption ionization–time of flight mass spectrometry (MALDI-TOF MS)-based identification were positive for Salmonella. According to the Kauffmann-White scheme (Institut Pasteur, Paris, France), the strain was serotyped as Salmonella enterica subsp. enterica serovar 1,3,19:nt.

Genomic DNA was extracted using the Wizard genomic DNA purification kit, according to the manufacturer’s protocol (Promega AG, Dübendorf, Switzerland). The genome was sequenced using two single-molecule real-time (SMRT) cells on a PacBio RS II (Pacific Biosciences, Menlo Park, CA, USA) and was performed at the Functional Genomics Center Zurich (Zurich, Switzerland). The raw reads were filtered using the RS_Filter_Only protocol in the SMRT Portal (Pacific Biosciences) with 0.75 as polymerase read quality cutoff and a minimal length of 500 bp. A total of 91,709 reads with an average length of 11,001 bp were selected, corresponding to 1,008,898,178 sequenced base pairs and 197-fold coverage. The reads were assembled using the Canu assembler 1.6 (1) with the option “pacbio-raw” and an estimated genome size of 5.1 Mbp. The Canu output consisted of 3 contigs, which were further polished in CLC Workbench 7 (CLC, Aarhus, Denmark). Two contigs were combined and formed the chromosomal sequence. The third contig contained a plasmid-like sequence. The start of the chromosome was set at the origin of replication, determined using Ori-Finder (2).

A blastN search with the complete chromosome against all Enterobacteriales genomes in the NCBI nucleotide database revealed a highest identity of 99% and a coverage of 91% with the genome of Salmonella enterica subsp. enterica serotype Senftenberg 775W (=ATCC 43845). Hence, strain N17-509 was classified as belonging to Salmonella enterica subsp. enterica serotype Senftenberg. The genome of N17-509 consists of a 5,121,989-bp chromosome and a 29,600-bp extrachromosomal element designated pN17-509. The GC contents of the chromosome and pN17-509 are 52.2% and 36.5%, respectively. The genome was annotated using the NCBI Prokaryotic Genomes Annotation Pipeline and contains 5,361 genes, of which 5,241 encode proteins. Further, 84 tRNA genes and 7 rRNA operons are present. Remarkably, the Salmonella pathogen island 1 (SPI-1), harboring, e.g., the *invA* gene, is absent in this strain. The plasmid pN17-509 has 97% identity at 87% coverage with the linear plasmid pBSSB1 from Salmonella enterica subsp. enterica serovar Typhi that mediates flagellar variation (3).

### Accession number(s).

The sequence and annotation data of the complete genome of Salmonella enterica subsp. enterica serovar Senftenberg strain N17-509 are deposited in the GenBank database with the accession numbers CP026379 for the chromosome and CP026380 for the plasmid.
